# Loss of Mll3 Catalytic Function Promotes Aberrant Myelopoiesis

**DOI:** 10.1371/journal.pone.0162515

**Published:** 2016-09-09

**Authors:** Kelly M. Arcipowski, Marinka Bulic, Sandeep Gurbuxani, Jonathan D. Licht

**Affiliations:** 1 Division of Hematology/Oncology, Northwestern University Feinberg School of Medicine, Chicago, Illinois, United States of America; 2 Department of Clinical Hematology/Hematopathology, University of Chicago Medical Center, Chicago, Illinois, United States of America; 3 The University of Florida Health Cancer Center, Gainesville, Florida, United States of America; Cincinnati Children's Hospital Medical Center, UNITED STATES

## Abstract

Two of the most common myeloid malignancies, myelodysplastic syndrome (MDS) and acute myeloid leukemia (AML), are associated with exceedingly low survival rates despite recent therapeutic advances. While their etiology is not completely understood, evidence suggests that certain chromosomal abnormalities contribute to MDS and AML progression. Among the most frequent chromosomal abnormalities in these disorders are alterations of chromosome 7: either complete loss of one copy of chromosome 7 (-7) or partial deletion of 7q (del(7q)), both of which increase the risk of progression from MDS to AML and are associated with chemoresistance. Notably, 7q36.1, a critical minimally deleted region in 7q, includes the gene encoding the histone methyltransferase mixed-lineage leukemia 3 (*MLL3*), which is also mutated in a small percentage of AML patients. However, the mechanisms by which *MLL3* loss contributes to malignancy are unknown. Using an engineered mouse model expressing a catalytically inactive form of Mll3, we found a significant shift in hematopoiesis toward the granulocyte/macrophage lineage, correlating with myeloid infiltration and enlargement of secondary lymphoid organs. Therefore, we propose that *MLL3* loss in patients may contribute to the progression of MDS and AML by promoting myelopoiesis.

## Introduction

Myelodysplastic syndrome (MDS) and acute myeloid leukemia (AML) are heterogeneous clonal disorders characterized by the failure of normal hematopoiesis and the accumulation of immature or incompletely differentiated myeloid precursors [1, 2]. MDS is associated with dysplasia in myeloid lineages, peripheral cytopenias, and intramedullary cell death [1, 3], while AML is defined by the accumulation of blasts (>20%) in the bone marrow (BM) [2]. MDS and AML are among the most common myeloid malignancies, with up to 40% of MDS patients developing AML [2, 4]. Despite recent advances in therapeutics, such as azacitidine for MDS [5], the long-term survival rates for most of these patients are poor.

Recent large-scale genomic sequencing studies of MDS and AML tumors revealed recurrent mutations in or deletions of epigenetic regulators [6–8]. Among such abnormalities is the loss of the histone methyltransferase mixed-lineage leukemia 3 (*MLL3*) due to complete loss of chromosome 7 (monosomy 7; -7) or 7q36.1 deletion (del(7q)) [9–12]. Monosomy 7 or del(7q) occurs in roughly 10% of *de novo* MDS, 50% of therapy-related MDS, and 7% of *de novo* AML cases [1, 2]. These chromosomal abnormalities are associated with an increased risk of AML development and worse prognosis due to enhanced disease progression and chemotherapeutic resistance [4, 10]. In addition to gene deletions, truncating mutations in *MLL3* are observed in approximately 1% of AML cases according to TCGA data [13–15] and other studies [6, 16]. The frequency of *MLL3* loss due to chromosome 7 aberrations and the poor prognosis of these patients implicate a potential role for MLL3 in the biology of MDS and AML. Loss-of-function mutations of *MLL3* are also common in other hematologic malignancies, such as multiple myeloma [17], as well as in solid tumors, including medulloblastoma [18], bladder [19], liver [20], gastric [21], pancreatic [22], prostate [23], ovarian [24], esophageal [25], colorectal [26], and breast cancers [27], suggesting an important role for MLL3 as a tumor suppressor.

MLL3 is a large protein of 4911 amino acids containing several important functional domains: the plant homeodomain (PHD) and FY-rich N-terminal (FYRN) domains that mediate protein-protein interactions, and the suppressor of variegation/enhancer of zeste/trithorax (SET) domain which confers histone 3 lysine 4 monomethyl (H3K4me1) catalytic activity associated with active enhancers [11, 28, 29]. The importance of enhancers has been underscored by the discovery of enhancer mutations in cancer, altering expression of linked genes [30–32].

A recent study showed that shRNA-mediated knockdown of *Mll3* and *Nf1* in *p53*-null murine hematopoietic progenitor cells generated AML upon transplantation into irradiated recipient mice [16]. These results are consistent with the notion that *MLL3* is a tumor suppressor in AML. However, the unique contributions of loss of MLL3 function to malignant hematopoiesis were not examined. Although the role of Mll3 has been characterized in nuclear receptor function [33–36], metabolism [35, 37], and circadian rhythm [38, 39], and loss of Mll3 catalytic activity is associated with the development of urothelial tumors [40], the functional role and importance of Mll3 in hematopoietic cell development/function has not been previously explored. We therefore studied the hematopoietic system in mice expressing a catalytically-inactive form of Mll3 [41], and found that loss of Mll3 catalytic function promoted myelopoiesis and myeloid infiltration into lymphoid organs, but was not sufficient to drive leukemia. Thus, results presented in this study reveal a novel role for Mll3 in the regulation of myelopoiesis and lymphoid organ structure.

## Materials and Methods

### Mice

*Mll3*^+/Δ^ mice, generated via a 61-amino acid in-frame deletion of exons 25 and 26 (encompassing the RYINHS catalytic core region of the SET domain), were a gift from Dr. Jae Lee (Oregon Health and Sciences University, Portland, OR) [41]. Previous studies maintained these mice on a 129SVJ x C57BL/6 background [34, 35, 37, 40–42] due to embryonic lethality of *Mll3*^Δ/Δ^ animals on a C57BL/6 background [37], but we maintained them on a C57BL/6 x ICR outbred background. Briefly, 129SVJ x C57BL/6 mice were backcrossed to C57BL/6 mice for four generations, bred with ICR mice, and pups were inbred for three generations. These mice were crossed to ICR mice, and pups were inbred for one generation (mice received). These mice were bred to C57BL/6 mice, and pups were inbred for one generation (current study). Mice were screened for mutant *Mll3* (S1 Fig) using these primers: 5’,5’-CGGGGTGTGTACATGTTCCGCATGGACAATGAC-3’; 3’,5’- TTCTCCTTTCTGTATCCTCCGGTTGGAGCTGAT-3’ (wild-type); and 5’,5’-GCCTGTATGCTGCTAGAGAC-3’; 3’,5’-CCTGCCTCTATTCAAAGATC-3’ (mutant). Mice were housed in a barrier facility, and procedures were performed as approved by the Northwestern University Institutional Animal Care and Use Committee (protocol # 2013–3123).

### Antibodies and Reagents

The following antibodies (Ab) and reagents were used: from BD Pharmingen (San Jose, CA)—APC-Cy7-labeled rat anti-mouse B220 (clone RA3-6B2), PE-Cy7-labeled rat anti-mouse CD8a (clone 53–6.7), PE-Cy7-labeled rat anti-mouse CD19 (clone 1D3), Alexa Fluor 647-labeled rat anti-mouse Mac1 (clone M1/70), FITC-labeled rat anti-mouse FcγR (clone 2.4G2), FITC-labeled hamster anti-mouse CD3e (clone 145-2C11), and rat anti-mouse FcγR blocking Ab (clone 2.4G2); from eBioscience (San Diego, CA)—eFluor 450-labeled rat anti-mouse CD4 (clone RM4-5), PerCP-Cy5.5-labeled rat anti-mouse Sca-1 (clone D7), eFluor 660-labeled rat anti-mouse CD34 (clone RAM34), PE-Cy7-labeled Streptavidin, and eFluor 450-labeled rat anti-mouse IL-7R (clone A7R34); from BD Biosciences (San Jose, CA)—PerCP-Cy5.5-labeled rat anti-mouse Gr1 (clone RB6-8C5); from BioLegend (San Diego, CA)—APC-Cy7-labeled rat anti-mouse c-kit (clone 2B8); and from Stemcell Technologies (Vancouver, BC, Canada)—EasySep Mouse Hematopoietic Progenitor Cell Isolation Cocktail (Lin).

### Flow Cytometry

BM (femurs and tibias), spleens, and cervical lymph nodes (LN) were harvested from 12-month-old mice. Erythrocytes were lysed (buffer: 4.1g NH_4_Cl, 0.5g KHCO_3_, 100mL 0.5M EDTA, H_2_O to 500mL), and 1 x 10^6^ cells were added to wells of a 96-well round-bottom plate in 50μL PBS containing 3% FBS (Life Technologies, Carlsbad, CA). Anti-mouse CD16/32 blocking Ab (0.5μg) was added 10 min before staining with fluorescently-labeled Abs (1μg). Flow cytometry was performed on an LSR II (BD, Franklin Lakes, NJ), and analyses were performed using FlowJo software (Tree Star, Ashland, OR).

### Histology

BM (sterna), spleens, and axillary/brachial LNs were harvested from 12-month-old mice. Paraffin processing, sectioning, Hematoxylin and Eosin (H&E) staining, and immunohistochemical (IHC) staining were performed by the Mouse Histology and Phenotyping Laboratory at Northwestern University.

### Microscopy

Images were taken using a Leica DM4000B microscope equipped with a Leica DFC320 camera and captured using Leica Application Suite V4.4 software (Leica Microsystems, Buffalo Grove, IL). Splenic follicles were measured via ImageJ.

### Cell Lines

Control (reference clone) and *MLL3*-targeted clones of the human chronic myeloid leukemia cell line KBM7, engineered via a gene-trap system, were purchased from Haplogen (Vienna, Austria) [43]. Cells were maintained in IMDM medium with 10% heat-inactivated fetal bovine serum (FBS) and antibiotics (Life Technologies, Grand Island, NY).

### qPCR

RNA was isolated from KBM7 cells using RNeasy Mini and QIAshredder kits (QIAGEN, Valencia, CA), and cDNA was made using the iScript cDNA Synthesis Kit (Bio-Rad, Hercules, CA), according to the manufacturer’s instructions. qPCR reactions were carried out using TaqMan Universal PCR Master Mix and associated probes recognizing *MLL3* (Applied Biosystems, Grand Island, NY) and run on a Roche LightCycler 480 II (Roche Diagnostics, Indianapolis, IN).

### Adhesion Assay

Adhesion of KBM7 cells to fibronectin was determined using the InnoCyte Fibronectin ECM Cell Adhesion Assay kit (Calbiochem, San Diego, CA). Briefly, 2 x 10^6^ cells were resuspended in 2mL serum-free medium per well in a 6-well plate. Calcein-AM solution was added to the cells at a concentration of 1μg/mL, and cells were incubated at 37°C for 1h. Cells were collected and washed with PBS and adhesion medium (for 50mL total volume: 49mL serum-free medium, 0.25g BSA, 1mL 1M HEPES), resuspended in adhesion medium at a concentration of 5 x 10^5^ cells/mL, and added to 6 wells of a 96-well fibronectin-coated plate, 100μL/well. Cells were then incubated at 37°C for 1h and washed with adhesion medium and PBS. Fluorescence was measured using a FLUOstar plate reader (BMG LABTECH Inc., Cary, NC).

### Migration Assay

2.5 x 10^4^ cells were resuspended in 150μL serum-free medium in 6.5mm Transwell Permeable Supports (Corning Inc., Lowell, MA) in a 24-well plate in triplicate. The wells of the plate contained 500μL of medium with 10% FBS. Wells containing serum-free medium were included as controls. After 24h, the medium in the bottom of the wells was collected, and cells were counted for 3 min/sample via flow cytometry. Flow cytometry was performed on an LSR II, and analyses were performed using FlowJo software to determine the number of migrating cells.

### Statistics

Analyses were performed with GraphPad Prism software (GraphPad Software, La Jolla, CA). Statistical comparisons were made using the Mann-Whitney or Student’s unpaired, two-sided *t*-test based upon whether data were normally distributed, as determined by the D'Agostino-Pearson and Shapiro-Wilk tests. *p* values <0.05 were considered statistically significant.

## Results

### Mll3 regulates marrow myeloid precursors

**Fig 1:**
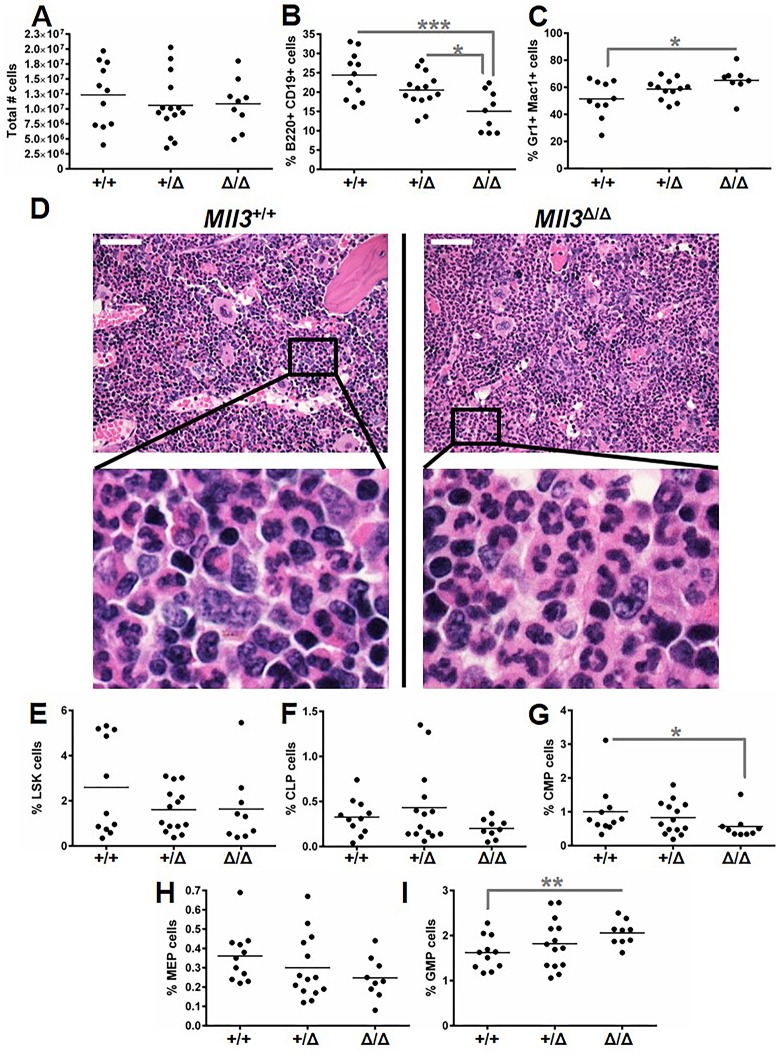
Impact of Mll3 loss-of-function on hematopoietic progenitor populations. Shown is **A**, the total number of BM cells, and the percentages of **B**, B cells and **C**, Gr1^+^ Mac1^+^ myeloid cells, where each point on the graph represents one mouse. Values in panels *B* and *C* are expressed as percentages of total cells. **D**, Representative H&E stains from one individual *Mll3*^+/+^ and *Mll3*^Δ/Δ^ mouse of sternum sections taken at 400X magnification. Images depict the entire field of view (top panels) and a zoomed-in view (bottom panels). Scale bars are 50μm. **E-I**, Similar to panels *B* and *C*, except that the percentages of LSK cells, CLP, CMP, MEP, and GMP are shown, respectively. **B, C, I**, *p<0.05, **p<0.01, ***p<0.005 as determined by the Student’s *t*-test. **G**, *p<0.05 as determined by the Mann-Whitney test.

The majority of *MLL3* mutations seen in AML patients are truncating mutations that result in loss of the catalytic SET domain [6, 16, 44]. To mimic the loss of MLL3 catalytic function in AML patients, we analyzed *Mll3*^Δ/Δ^ mice expressing a mutant form of Mll3 in which two exons in the catalytic SET domain have been deleted without alteration of protein or transcript levels ([41] and S1 Fig). As these diseases both result from abnormal hematopoiesis and carry an increased incidence with age [1, 2], we investigated the effects of Mll3 loss-of-function on mature and progenitor populations in the BM in a cohort of aged mice (see S2 and S3 Figs for gating strategy). Previous studies described a reduced Mendelian frequency of *Mll3*^Δ/Δ^ mice on a 129SVJ x C57BL/6 background [41], as well as stunted growth [41], likely due to the metabolic aberrations and defective adipogenesis in these animals [35, 37]. Therefore, mice in this study were maintained on a C57BL/6 x ICR background to increase the number of viable *Mll3*^Δ/Δ^ animals as a result of the larger litter sizes characteristic of the partially outbred ICR genetic background. Despite normal overall BM cellularity (Fig 1A), *Mll3*^Δ/Δ^ BM exhibited a significant decrease in B cells and a substantial increase in Gr1^+^ Mac1^+^ cells compared to *Mll3*^+/+^ mice (Fig 1B and 1C). This enhanced granulopoiesis was also evident in histological BM sections, with BM from *Mll3*^Δ/Δ^ mice showing increased numbers of granulocytes displaying the characteristic doughnut-shaped or multi-lobed nuclei (Fig 1D and S4 Fig). Further examination of *Mll3*^Δ/Δ^ BM stem and progenitor populations revealed a trending decrease in Lin^-^/Sca-1^+^/ckit^+^ cells (LSKs) and common lymphoid progenitors (CLPs) (Fig 1E and 1F), and a clear significant decrease in common myeloid progenitors (CMPs) (Fig 1G). There was also a trending decrease in megakaryocyte/erythroid progenitors (MEPs) compared to *Mll3*^+/+^ BM (Fig 1H). However, *Mll3*^Δ/Δ^ granulocyte/macrophage progenitors (GMPs) were significantly increased (Fig 1I), correlating with the observed accumulation of Gr1^+^ Mac1^+^ cells. Therefore, the expansion of myeloid cells in the BM likely resulted from enhanced myelopoiesis at the GMP stage.

**Fig 2:**
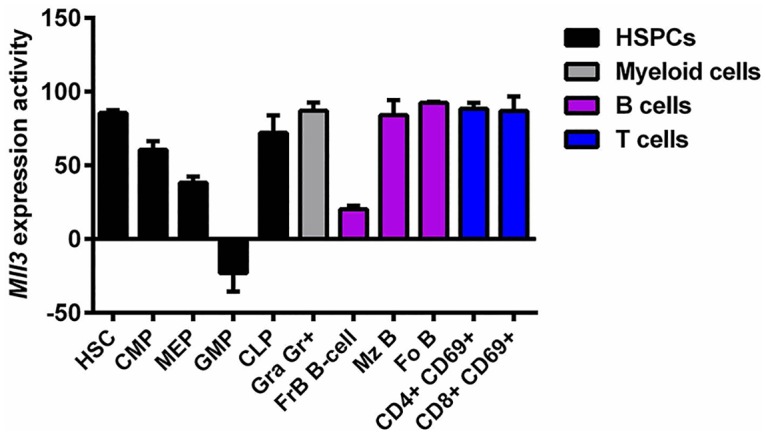
Relative *Mll3* expression in mouse hematopoietic cells. Absolute gene expression profiling adapted from Gene Expression Commons evaluating *Mll3* expression across 39 different hematopoietic cell populations in the mouse BM, spleen, and thymus. Shown are the normalized values of *Mll3* expression activity for several cell populations from 2–4 biological replicates. Positive values indicate high *Mll3* expression compared to the common reference, and negative values denote relatively low *Mll3* expression in comparison to the common reference. Error bars represent mean + SD. HSC, hematopoietic stem cell; Gra, granulocyte; Fr, fraction; Mz, marginal zone; Fo, follicular.

To determine an underlying genetic explanation for the increase in GMPs, we examined data from Gene Expression Commons [45]. These data revealed that although *Mll3* is highly expressed in nearly all hematopoietic cell types of both the myeloid and lymphoid lineages, *Mll3* expression is appreciably lower in GMPs compared to the common reference, a pool of ~12,000 publically available Affymetrix mouse 430 2.0 microarray data (Fig 2 and S5 Fig). These data suggest that GMPs may be less dependent on *Mll3* and that loss of Mll3 function would favor the development and growth of this cell population. Although granulocytes express high levels of *Mll3*, it is possible that Mll3 catalytic function is not required for the development of these cells, which is what our data suggest. Taken together, these results show that loss of Mll3 function shifts hematopoiesis in favor of the granulocyte/macrophage lineage.

### Loss of Mll3 catalytic function leads to splenomegaly and lymphadenopathy

**Fig 3:**
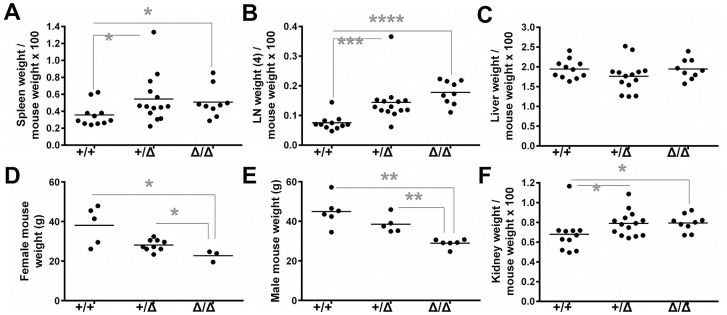
Phenotypic characterization of mutant *Mll3* mice. **A**, Spleen weight expressed as a percentage of total mouse weight and **B**, the combined weight of four LN normalized to total mouse weight. Each point represents one mouse, and graphs include a combination of female and male organ weights. **C**, Similar to panel *A*, except liver weight is shown. **D**, Female mouse weight, where each point represents one mouse. **E**, Similar to panel *D*, except male mouse weight is shown. **F**, Similar to panels *A* and *C*, except kidney weight is shown. *p<0.05, **p<0.005, ***p<0.0005, ****p<0.0001 as determined by the Mann-Whitney test.

Although *Mll3*^Δ/Δ^ mice displayed a myelopoietic phenotype in the BM, these animals did not develop MDS or AML, as measured by peripheral blood analysis. We did not observe any changes in the number of white blood cells, neutrophils/granulocytes, monocytes, red blood cells, or platelets between *Mll3*^+/+^ and *Mll3*^Δ/Δ^ mice (S6 Fig). Thus, we next examined *Mll3*^Δ/Δ^ mice to determine if the myeloid bias observed in the BM manifested in organs commonly associated with myeloid infiltration in MDS and AML. A frequent observation in patients with myeloid malignancy is enlargement of secondary lymphoid organs due to infiltration and accumulation of tumor cells [16, 46]. Interestingly, although none of the aged mice developed hematologic tumors, *Mll3*^Δ/Δ^ mice displayed splenomegaly and lymphadenopathy (Fig 3A and 3B). These results suggest an important role for Mll3 in the regulation of hematopoietic cells in secondary lymphoid organs. Given the important role of Mll3 in liver X receptor signaling and liver adipogenesis [34, 37], and that the liver is a common site of leukemic infiltration in AML [46], we also examined the liver. However, no significant differences in liver weight were observed among *Mll3*^+/+^, *Mll3*^+/Δ^, and *Mll3*^Δ/Δ^ mice (Fig 3C), consistent with the lack of disease development in *Mll3*^Δ/Δ^ mice.

Previous reports describe urological and metabolic abnormalities in these *Mll3*^Δ/Δ^ mice. Thus, we wanted to determine if the substantial hematopoietic phenotype we saw had any impact on the major phenotypes noted in these prior studies. As in previous reports, we observed a reduction in the frequency of homozygous mutant animals, as well as significantly lower weight of *Mll3*^Δ/Δ^ mice compared to *Mll3*^+/+^ mice (Fig 3D and 3E). Although one study reported spontaneous urothelial tumors in *Mll3*^Δ/Δ^ mice [40], we did not observe such tumors in the ICR-crossed strain. However, *Mll3*^Δ/Δ^ mice did have significantly enlarged kidneys (Fig 3F), correlating with the previously reported hydronephrosis, expanded renal pelvic space, and expanded interstitial compartment of *Mll3*^Δ/Δ^ kidneys [40]. In conclusion, aged *Mll3*^Δ/Δ^ mice did not develop MDS or AML, but loss of Mll3 catalytic function recapitulated some previously reported phenotypes and resulted in the novel finding of splenomegaly and lymphadenopathy.

### Loss of Mll3 catalytic function alters lymphoid organ composition

**Fig 4:**
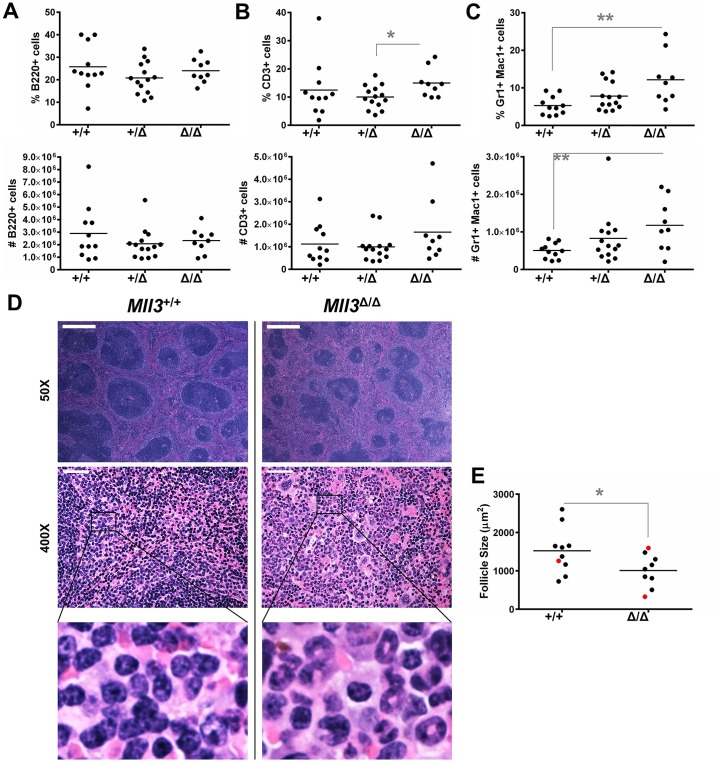
Role of Mll3 in immune cell composition of the spleen. The percentage of total cells (top) and absolute number (bottom) of **A**, B cells, **B**, T cells, and **C**, Gr1^+^ Mac1^+^ myeloid cells is shown, where each point represents one mouse. **D**, Representative H&E stains of spleen sections (50X magnification, top panels; 400X magnification, middle panels) from one individual *Mll3*^+/+^ and *Mll3*^Δ/Δ^ mouse. Images in the bottom panels are zoomed-in views of the middle panels. Scale bars are 500μm (top panels) and 50μm (middle panels). **E**, Quantification of splenic follicle size. Each point on the graph represents the average follicle size (black, ≥10 follicles measured; red, <10 follicles measured) for one mouse. *p<0.05, **p<0.01 as determined by the Student’s *t*-test.

The observed alterations in spleen and LN size, as well as BM hematopoiesis, prompted us to further examine secondary lymphoid organs to determine if the alterations seen in the BM manifested in the spleen and LNs, accounting for their increased size. Although the proportions of splenic T cells and Gr1^+^ Mac1^+^ cells (see S7 Fig for gating strategy) in *Mll3*^Δ/Δ^ mice were significantly altered compared to *Mll3*^+/+^ mice, the total B and T cell numbers remained relatively unchanged (Fig 4A, 4B and 4C). Rather, this difference in proportions was due to a significant accumulation of Gr1^+^ Mac1^+^ cells (Fig 4C). This myeloid expansion may have contributed to the observed splenomegaly in *Mll3*^Δ/Δ^ animals (Fig 3A). Histological examination revealed significantly smaller lymphoid follicles in the spleens of *Mll3*^Δ/Δ^ mice compared to *Mll3*^+/+^ mice (Fig 4D, 4E and S8 Fig). This aberrant architecture may be due in part to the increased number of myeloid cells in *Mll3*^Δ/Δ^ mice, as seen in histological sections of the spleen (Fig 4D).

**Fig 5:**
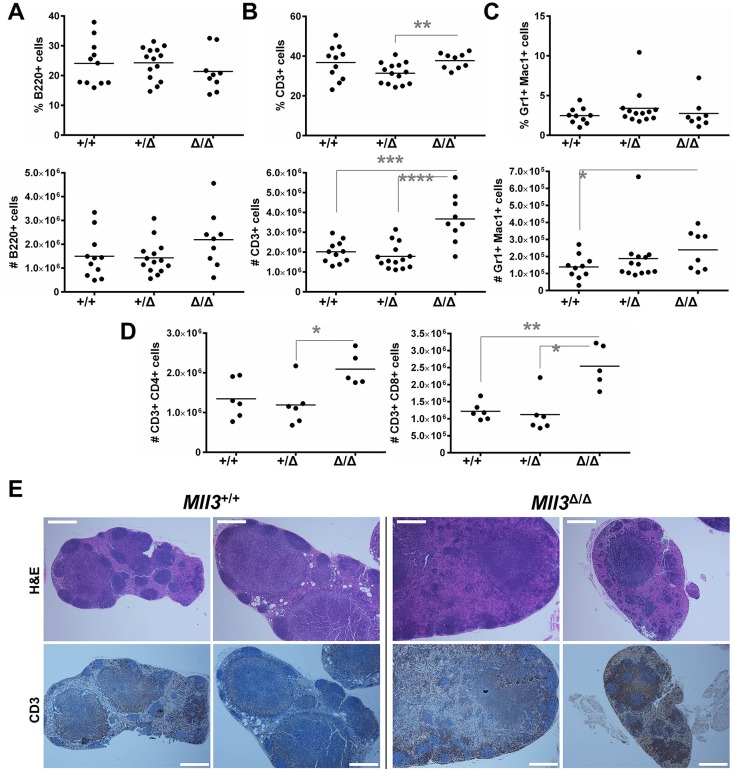
Quantification and distribution of immune cells in the LNs of *Mll3* mice. Shown is the percentage of total cells (top) and absolute number (bottom) of **A**, B cells, **B**, T cells, and **C**, Gr1^+^ Mac1^+^ myeloid cells. **D**, Similar to panels *A-C*, except the total numbers of CD4^+^ (left) and CD8^+^ (right) T cells are shown. Each point represents one mouse. **B-C**, *p<0.05, **p<0.01, ***p<0.001, ****p<0.0001 as determined by the Student’s *t*-test. **D**, *p<0.05, **p<0.005 as determined by the Mann-Whitney test. **E**, Representative H&E stains (top panels) and IHC for CD3 (bottom panels) from two individual *Mll3*^+/+^ and *Mll3*^Δ/Δ^ mice of LN sections taken at 50X magnification. Scale bars are 500μm.

Unlike our observations in the spleen, loss of Mll3 function did not significantly alter the proportions of *Mll3*^Δ/Δ^ lymphoid and myeloid cells in the LN (Fig 5A, 5B and 5C). However, *Mll3*^Δ/Δ^ LNs contained a significantly higher number of T cells and Gr1^+^ Mac1^+^ cells (see S9 Fig for gating strategy) compared to *Mll3*^+/+^ LNs (Fig 5B and 5C). T cell accumulation was not due to alterations in any one subset, as both CD4^+^ and CD8^+^ T cells were increased (Fig 5D). Histological analysis revealed that Mll3 loss-of-function significantly altered LN architecture and size, affecting follicle distribution/size and resulting in large areas of T cell accumulation (Fig 5E and S10 Fig). Thus, we conclude that myeloid cell expansion contributed to the splenomegaly/lymphadenopathy in *Mll3*^Δ/Δ^ mice, and disrupted normal secondary lymphoid architecture.

### *MLL3* disruption leads to increased cell adhesion and migration *in vitro*

**Fig 6:**
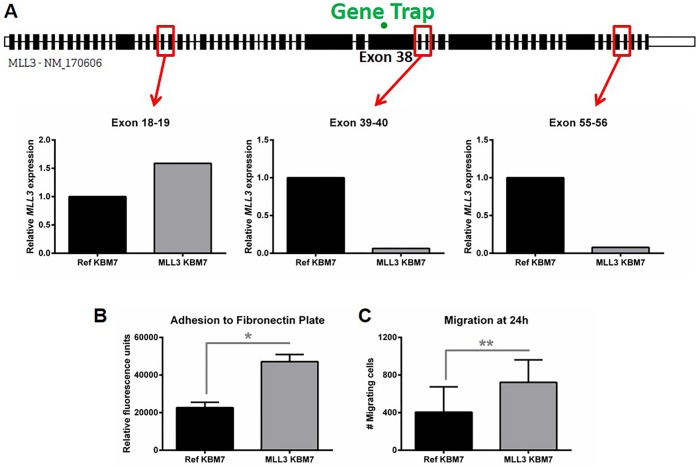
Effects of *MLL3* disruption on myeloid cell adhesion and migration. **A**, Relative *MLL3* expression in human KBM7 cells as determined by qPCR. TaqMan probes (indicated in red) recognized exon junctions before (exons 18–19) and after (exons 39–40 and 55–56) the gene-trap (exon 38), indicated in green. **B**, Relative adhesion of KBM7 cells to fibronectin as measured in fluorescence units. Shown is the average of technical replicates from four individual experiments + SEM. **C**, The number of KBM7 cells that migrated across a trans-well membrane in response to FBS after 24h. Data are the combination of technical replicates from four individual experiments + SD. Ref, control gene-trap; MLL3, *MLL3*-targeted gene-trap. *p<0.05, **p<0.01 as determined by the Mann-Whitney test.

The significant expansion of myeloid cells in secondary lymphoid organs (Figs 4 and 5), but lack of myeloid cell accumulation in the peripheral blood (S6 Fig) upon loss of Mll3 function suggested a defect in cellular migration. Using a human myeloid leukemia cell line containing a gene trap to delete the catalytic SET domain of MLL3 (Fig 6A), we found a significant increase in cell adhesion to the blood plasma and extracellular matrix constituent fibronectin (Fig 6B), as well as significantly enhanced cell migration in response to serum (Fig 6C) upon loss of MLL3 function. Thus, altered cell migration and adhesion may account for the myeloid accumulation in secondary lymphoid organs *in vivo* (Figs 4 and 5).

## Discussion

*MLL3* is commonly lost in myeloid malignancies due to chromosome 7 alterations [9–12] and has been implicated as a tumor suppressor in AML [16]. However, its role in normal hematopoiesis was unknown. Our study demonstrates that loss of Mll3 histone methyltransferase activity led to an expansion of GMPs at the expense of MEPs and CMPs (Fig 1). This finding mirrors what is observed in high-risk MDS and AML patients, who exhibit similar alterations of progenitor populations [47]. GMP cells express very little *Mll3*, especially compared to other hematopoietic cell types (Fig 2 and S5 Fig). Thus, loss of MLL3 may promote the transition from MDS to AML by shifting hematopoiesis in favor of the apparent Mll3-independent GMPs, which give rise to malignancy. In our mouse model of mutant *Mll3*, loss of catalytic function alone was not sufficient to drive malignancy (S6 Fig), even though loss of *MLL3* due to chromosome 7 aberrations is associated with increased risk of AML development and poor prognosis [4, 11]. Collectively, these results suggest that while MLL3 loss alone is not sufficient for malignancy, it may be necessary to drive disease progression in a subset of patients. This idea is supported by a recent study showing that Mll3 serves as a haploinsufficient tumor suppressor in AML, and that *Mll3* knockdown cooperates with *p53* and/or *Nf1* loss to accelerate disease [16].

The increased myelopoiesis in the BM of *Mll3*^Δ/Δ^ mice translated into the accumulation of Gr1^+^ Mac1^+^ cells in both the spleen and LNs, likely accounting for the observed splenomegaly and lymphadenopathy (Figs 3, 4 and 5). Further histological examination of secondary lymphoid architecture revealed disruption of normal follicle formation, likely due to overcrowding by infiltrating myeloid cells, as B cell numbers were not decreased in the spleen and LNs upon loss of Mll3 function (Figs 4 and 5). However, there are other cell populations that may also contribute to the increased spleen and LN size, such as dendritic cells, natural killer cells, and stromal cells. As Mll3 is ubiquitously expressed in the *Mll3*^Δ/Δ^ animals [41] and in many different hematopoietic cells (Fig 2 and S5 Fig), Mll3 may play an important role in these other cell types as well. Finally, we observed a significant increase in total T cell numbers in the LNs of *Mll3*^Δ/Δ^ mice (Fig 5), without a concurrent increase in splenic T cell numbers (Fig 4) and no significant change in CLPs in the bone marrow (Fig 1). This suggests that the expansion of T cells in the LN is most likely due to increased T cell proliferation. As *Mll3* expression is high in T cells compared to other cell types (Fig 2), it is possible that Mll3 negatively regulates T cell proliferation, such that reduced Mll3 activity leads to T cell expansion. Furthermore, Gr1^+^ Mac1^+^ macrophages/monocytes are well-known inducers of T cell proliferation, and *Mll3*^Δ/Δ^ mice have a significant expansion of these cells in secondary lymphoid organs (Figs 4 and 5), which may contribute to the observed increase in T cell numbers.

Gr1^+^ Mac1^+^ expansion in *Mll3*^Δ/Δ^ mice may be due to altered hematopoiesis, as we observed a significant increase in the BM precursor GMP population (Fig 1). However, this expansion may also be due to enhanced cell proliferation, survival, or homing, the latter of which may explain why we did not observe an increase in peripheral neutrophils in *Mll3*^Δ/Δ^ mice (S6 Fig). This idea is further supported by the enhanced cellular adhesion and migration observed in human cell lines upon loss of MLL3 function (Fig 6). However, the myeloid and T cell expansion (Fig 5) may also reflect inflammation, which is critical for normal immunity as well as disease progression. For example, myeloid-derived suppressor cell, regulatory T cell, and autoreactive CD8^+^ T cell accumulation is seen in MDS patients, correlating with immune suppression and autoimmunity, which is thought to contribute to BM failure in MDS [1, 48]. In this way, loss of MLL3 may contribute to disease development by reducing anti-tumor immune responses and/or contributing to autoimmunity, leading to cytokine-mediated intramedullary apoptosis and cytopenia [1, 48, 49]. This idea has precedent, as recent studies revealed epigenetic dysregulation in numerous autoimmune diseases [50], implicating an important role for epigenetics in regulating immune homeostasis.

*MLL3* is commonly lost in MDS and AML via 7q36.1 deletion, which carries a poor prognosis [4, 9–12]. Understanding the exact mechanisms by which MLL3 regulates normal hematopoiesis/immunity and promotes tumorigenesis may be useful for designing therapies that target the pathways affected by pathological *MLL3* loss, as controlled restoration of MLL3 itself may not be feasible. Our data demonstrate an important role for Mll3 in the regulation of myelopoiesis, suggest several avenues by which loss of MLL3 may contribute to MDS/AML development or progression, and represent the first investigation into the role of Mll3 in hematopoiesis *in vivo*. As the overall prognosis of MDS/AML associated with chromosome 7 abnormalities is poor, further delineating how *MLL3* loss promotes malignancy may identify possible new pathways for improved therapy.

## Supporting Information

S1 Fig *Mll3* mouse genotyping and expression in hematopoietic cells. **(Top)**, Tail DNA was analyzed by PCR using primers recognizing either the wild-type allele (+) or the mutant allele (Δ). Products were resolved on an agarose gel, and images were obtained using a Syngene U:Genius3 imaging system (Syngene USA, Frederick, MD). Shown are representative images (top panel, + allele; bottom panel, Δ allele) of specimens used in this study. C, control; -C, no template water control. **(Bottom)**, BM (left) and spleens (right) were harvested from 12-month-old *Mll3*^+/+^ and *Mll3*^Δ/Δ^ mice. Cells were either lineage-depleted (BM) using the EasySep Mouse Hematopoietic Progenitor Cell Isolation Kit, or enriched for Gr1^+^ cells (spleen) using the EasySep Mouse Neutrophil Enrichment Kit (Stemcell Technologies). qPCR was performed to determine the relative levels of mutant *Mll3* expression, normalized to wild-type *Mll3*, using the following primers: 5’,5’-GTGGCAGAGGCAGGTCTAAA-3’; 3’,5’-GCTGCAAAGTGAACTTGTCACTAC-3’. Left, *n* = 4 per group; right, *n* = 1 (*Mll3*^+/+^), *n* = 3 (*Mll3*^Δ/Δ^).
(PDF)

S2 Fig Flow cytometric analysis of hematopoietic stem and progenitor cells in the BM. Representative FACS plots are shown for *Mll3*^+/+^, *Mll3*^+/Δ^, and *Mll3*^Δ/Δ^ mice. Cells were gated on the Lin^-^ compartment, and then categorized based on c-kit and Sca-1 expression as LSK cells (c-kit^+^ Sca-1^+^), CLP cells (c-kit^int^ Sca-1^-^ IL-7R^+^), or myeloid progenitor cells (c-kit^+^ Sca-1^-^), which were further classified as MEP cells (CD34^-^ FcγR^-^), CMP cells (CD34^+^ FcγR^-^), or GMP cells (CD34^+^ FcγR^+^).
(PDF)

S3 Fig Gating strategy for analysis of BM B cells and myeloid cells by flow cytometry. Shown are representative FACS plots for *Mll3*^+/+^, *Mll3*^+/Δ^, and *Mll3*^Δ/Δ^ mice. Cells were gated according to CD19 and B220 expression, where B cells were considered positive for both markers, or according to expression of Gr1 and Mac1 (myeloid cells).
(PDF)

S4 Fig Histological analysis of BM from *Mll3*^+/Δ^ mice. Representative H&E stains from two individual *Mll3*^+/Δ^ mice of sternum sections taken at 400X magnification. Images show the entire field of view (top panels) and a zoomed-in view (bottom panels). Scale bars are 50μm. Compare to Fig 1D.
(PDF)

S5 Fig Model of gene expression profiling adapted from Gene Expression Commons. Chart depicts *Mll3* expression across 39 hematopoietic cell populations in the mouse BM, spleen, and thymus. Pink denotes populations with high *Mll3* expression compared to the common reference, whereas blue indicates populations with relatively low *Mll3* expression. HSC, hematopoietic stem cell; MPP, multi-potent progenitor; GMLP, granulocyte/macrophage/lymphoid progenitor; MkP, megakaryocyte progenitor; Gra, granulocyte; Mono, monocyte; BLP, B-lymphoid progenitor; Fr, fraction; Mz, marginal zone; Fo, follicular; NK, natural killer cell; DN, double negative; DP, double positive; Plt, platelet; Ery, erythrocyte.
(PDF)

S6 Fig Complete blood cell count analysis in *Mll3* mice. Blood samples were collected via tail nick from 12-month-old mice and run on a Hemavet 950 FS (Drew Scientific, Inc., Miami Lakes, FL) to obtain blood cell counts. Shown is the number of **A**, white blood cells, **B**, neutrophils/granulocytes, **C**, monocytes, **D**, red blood cells, and **E**, platelets, where each point represents one mouse. *p<0.05 as determined by the Student’s *t*-test.
(PDF)

S7 Fig Analysis of splenic lymphoid and myeloid cells by flow cytometry. Representative FACS plots are shown for *Mll3*^+/+^, *Mll3*^+/Δ^, and *Mll3*^Δ/Δ^ mice. Cells were categorized according to expression of B220 (B cells), CD3 (T cells), or Gr1 and Mac1 (myeloid cells).
(PDF)

S8 Fig Splenic architecture in heterozygous *Mll3* mice. Representative H&E stains of spleen sections taken at 50X magnification from four individual *Mll3*^+/Δ^ mice. Scale bars are 500μm. Compare to Fig 4D.
(PDF)

S9 Fig Flow cytometric characterization of lymphoid subsets and myeloid cells in the LN. Representative FACS plots for *Mll3*^+/+^, *Mll3*^+/Δ^, and *Mll3*^Δ/Δ^ mice. Cells were classified according to expression of B220 (B cells), CD3 (T cells), or Gr1 and Mac1 (myeloid cells). CD3^+^ cells were further categorized into T cell subsets based on expression of CD4 and CD8.
(PDF)

S10 Fig Histological examination of *Mll3*^+/Δ^ LNs. Representative H&E stains from four individual *Mll3*^+/Δ^ mice of LN sections (50X magnification). Scale bars are 500μm. Compare to Fig 5E.
(PDF)

## References

[1] Visconte V, Tiu RV, Rogers HJ. Pathogenesis of myelodysplastic syndromes: an overview of molecular and non-molecular aspects of the disease. Blood Res. 2014;49(4):216–27. Epub 2014/12/31. 10.5045/br.2014.49.4.216 25548754 PMC4278002

[2] Estey E, Dohner H. Acute myeloid leukaemia. Lancet. 2006;368(9550):1894–907. Epub 2006/11/28. 17126723 10.1016/S0140-6736(06)69780-8

[3] Shetty V, Hussaini S, Broady-Robinson L, Allampallam K, Mundle S, Borok R, et al Intramedullary apoptosis of hematopoietic cells in myelodysplastic syndrome patients can be massive: apoptotic cells recovered from high-density fraction of bone marrow aspirates. Blood. 2000;96(4):1388–92. Epub 2000/08/15. 10942382

[4] Pellagatti A, Cazzola M, Giagounidis A, Perry J, Malcovati L, Della Porta MG, et al Deregulated gene expression pathways in myelodysplastic syndrome hematopoietic stem cells. Leukemia. 2010;24(4):756–64. Epub 2010/03/12. 10.1038/leu.2010.31 20220779

[5] Fenaux P, Mufti GJ, Hellstrom-Lindberg E, Santini V, Finelli C, Giagounidis A, et al Efficacy of azacitidine compared with that of conventional care regimens in the treatment of higher-risk myelodysplastic syndromes: a randomised, open-label, phase III study. Lancet Oncol. 2009;10(3):223–32. Epub 2009/02/24. 10.1016/S1470-2045(09)70003-8 19230772 PMC4086808

[6] Dolnik A, Engelmann JC, Scharfenberger-Schmeer M, Mauch J, Kelkenberg-Schade S, Haldemann B, et al Commonly altered genomic regions in acute myeloid leukemia are enriched for somatic mutations involved in chromatin remodeling and splicing. Blood. 2012;120(18):e83–92. Epub 2012/09/15. 10.1182/blood-2011-12-401471 22976956

[7] Nikoloski G, Langemeijer SM, Kuiper RP, Knops R, Massop M, Tonnissen ER, et al Somatic mutations of the histone methyltransferase gene EZH2 in myelodysplastic syndromes. Nat Genet. 2010;42(8):665–7. Epub 2010/07/06. 10.1038/ng.620 20601954

[8] Zhang L, Padron E, Lancet J. The molecular basis and clinical significance of genetic mutations identified in myelodysplastic syndromes. Leuk Res. 2015;39(1):6–17. Epub 2014/12/04. 10.1016/j.leukres.2014.10.006 25465125

[9] Kuhn MW, Radtke I, Bullinger L, Goorha S, Cheng J, Edelmann J, et al High-resolution genomic profiling of adult and pediatric core-binding factor acute myeloid leukemia reveals new recurrent genomic alterations. Blood. 2012;119(10):e67–75. Epub 2012/01/12. 10.1182/blood-2011-09-380444 22234698 PMC3311263

[10] Dohner K, Brown J, Hehmann U, Hetzel C, Stewart J, Lowther G, et al Molecular cytogenetic characterization of a critical region in bands 7q35-q36 commonly deleted in malignant myeloid disorders. Blood. 1998;92(11):4031–5. Epub 1998/12/03. 9834205

[11] Ruault M, Brun ME, Ventura M, Roizes G, De Sario A. MLL3, a new human member of the TRX/MLL gene family, maps to 7q36, a chromosome region frequently deleted in myeloid leukaemia. Gene. 2002;284(1–2):73–81. Epub 2002/03/14. 11891048 10.1016/s0378-1119(02)00392-x

[12] Kotini AG, Chang CJ, Boussaad I, Delrow JJ, Dolezal EK, Nagulapally AB, et al Functional analysis of a chromosomal deletion associated with myelodysplastic syndromes using isogenic human induced pluripotent stem cells. Nat Biotechnol. 2015;33(6):646–55. Epub 2015/03/24. 10.1038/nbt.3178 25798938 PMC4464949

[13] Gao J, Aksoy BA, Dogrusoz U, Dresdner G, Gross B, Sumer SO, et al Integrative analysis of complex cancer genomics and clinical profiles using the cBioPortal. Sci Signal. 2013;6(269):pl1 Epub 2013/04/04. 10.1126/scisignal.2004088 23550210 PMC4160307

[14] Cerami E, Gao J, Dogrusoz U, Gross BE, Sumer SO, Aksoy BA, et al The cBio cancer genomics portal: an open platform for exploring multidimensional cancer genomics data. Cancer Discov. 2012;2(5):401–4. Epub 2012/05/17. 10.1158/2159-8290.CD-12-0095 22588877 PMC3956037

[15] Genomic and epigenomic landscapes of adult de novo acute myeloid leukemia. N Engl J Med. 2013;368(22):2059–74. Epub 2013/05/03. 10.1056/NEJMoa1301689 23634996 PMC3767041

[16] Chen C, Liu Y, Rappaport AR, Kitzing T, Schultz N, Zhao Z, et al MLL3 is a haploinsufficient 7q tumor suppressor in acute myeloid leukemia. Cancer Cell. 2014;25(5):652–65. Epub 2014/05/06. 10.1016/j.ccr.2014.03.016 24794707 PMC4206212

[17] Chapman MA, Lawrence MS, Keats JJ, Cibulskis K, Sougnez C, Schinzel AC, et al Initial genome sequencing and analysis of multiple myeloma. Nature. 2011;471(7339):467–72. Epub 2011/03/25. 10.1038/nature09837 21430775 PMC3560292

[18] Parsons DW, Li M, Zhang X, Jones S, Leary RJ, Lin JC, et al The genetic landscape of the childhood cancer medulloblastoma. Science. 2011;331(6016):435–9. Epub 2010/12/18. 10.1126/science.1198056 21163964 PMC3110744

[19] Gui Y, Guo G, Huang Y, Hu X, Tang A, Gao S, et al Frequent mutations of chromatin remodeling genes in transitional cell carcinoma of the bladder. Nat Genet. 2011;43(9):875–8. Epub 2011/08/09. 10.1038/ng.907 21822268 PMC5373841

[20] Fujimoto A, Totoki Y, Abe T, Boroevich KA, Hosoda F, Nguyen HH, et al Whole-genome sequencing of liver cancers identifies etiological influences on mutation patterns and recurrent mutations in chromatin regulators. Nat Genet. 2012;44(7):760–4. Epub 2012/05/29. 10.1038/ng.2291 22634756

[21] Zang ZJ, Cutcutache I, Poon SL, Zhang SL, McPherson JR, Tao J, et al Exome sequencing of gastric adenocarcinoma identifies recurrent somatic mutations in cell adhesion and chromatin remodeling genes. Nat Genet. 2012;44(5):570–4. Epub 2012/04/10. 10.1038/ng.2246 22484628

[22] Biankin AV, Waddell N, Kassahn KS, Gingras MC, Muthuswamy LB, Johns AL, et al Pancreatic cancer genomes reveal aberrations in axon guidance pathway genes. Nature. 2012;491(7424):399–405. Epub 2012/10/30. 10.1038/nature11547 23103869 PMC3530898

[23] Lindberg J, Mills IG, Klevebring D, Liu W, Neiman M, Xu J, et al The mitochondrial and autosomal mutation landscapes of prostate cancer. Eur Urol. 2013;63(4):702–8. Epub 2012/12/26. 10.1016/j.eururo.2012.11.053 23265383

[24] Kanchi KL, Johnson KJ, Lu C, McLellan MD, Leiserson MD, Wendl MC, et al Integrated analysis of germline and somatic variants in ovarian cancer. Nat Commun. 2014;5:3156 Epub 2014/01/23. 10.1038/ncomms4156 24448499 PMC4025965

[25] Gao YB, Chen ZL, Li JG, Hu XD, Shi XJ, Sun ZM, et al Genetic landscape of esophageal squamous cell carcinoma. Nat Genet. 2014;46(10):1097–102. Epub 2014/08/26. 10.1038/ng.3076 25151357

[26] Ashktorab H, Schaffer AA, Daremipouran M, Smoot DT, Lee E, Brim H. Distinct genetic alterations in colorectal cancer. PloS One. 2010;5(1):e8879 Epub 2010/02/04. 10.1371/journal.pone.0008879 20126641 PMC2811180

[27] Ellis MJ, Ding L, Shen D, Luo J, Suman VJ, Wallis JW, et al Whole-genome analysis informs breast cancer response to aromatase inhibition. Nature. 2012;486(7403):353–60. Epub 2012/06/23. 10.1038/nature11143 22722193 PMC3383766

[28] Herz HM, Mohan M, Garruss AS, Liang K, Takahashi YH, Mickey K, et al Enhancer-associated H3K4 monomethylation by Trithorax-related, the Drosophila homolog of mammalian Mll3/Mll4. Genes Dev. 2012;26(23):2604–20. Epub 2012/11/21. 10.1101/gad.201327.112 23166019 PMC3521626

[29] Hu D, Gao X, Morgan MA, Herz HM, Smith ER, Shilatifard A. The MLL3/MLL4 branches of the COMPASS family function as major histone H3K4 monomethylases at enhancers. Mol Cell Biol. 2013;33(23):4745–54. Epub 2013/10/02. 10.1128/MCB.01181-13 24081332 PMC3838007

[30] Mansour MR, Abraham BJ, Anders L, Berezovskaya A, Gutierrez A, Durbin AD, et al Oncogene regulation. An oncogenic super-enhancer formed through somatic mutation of a noncoding intergenic element. Science. 2014;346(6215):1373–7. Epub 2014/11/15. 10.1126/science.1259037 25394790 PMC4720521

[31] Puente XS, Bea S, Valdes-Mas R, Villamor N, Gutierrez-Abril J, Martin-Subero JI, et al Non-coding recurrent mutations in chronic lymphocytic leukaemia. Nature. 2015. Epub 2015/07/23. 10.1038/nature14666 26200345

[32] Herz HM, Hu D, Shilatifard A. Enhancer malfunction in cancer. Mol Cell. 2014;53(6):859–66. Epub 2014/03/25. 10.1016/j.molcel.2014.02.033 24656127 PMC4049186

[33] Lee S, Kim DH, Goo YH, Lee YC, Lee SK, Lee JW. Crucial roles for interactions between MLL3/4 and INI1 in nuclear receptor transactivation. Mol Endocrinol. 2009;23(5):610–9. Epub 2009/02/18. 10.1210/me.2008-0455 19221051 PMC2675954

[34] Lee S, Lee J, Lee SK, Lee JW. Activating signal cointegrator-2 is an essential adaptor to recruit histone H3 lysine 4 methyltransferases MLL3 and MLL4 to the liver X receptors. Mol Endocrinol. 2008;22(6):1312–9. Epub 2008/03/29. 10.1210/me.2008-0012 18372346 PMC2422828

[35] Kim DH, Lee J, Lee B, Lee JW. ASCOM controls farnesoid X receptor transactivation through its associated histone H3 lysine 4 methyltransferase activity. Mol Endocrinol. 2009;23(10):1556–62. Epub 2009/06/27. 10.1210/me.2009-0099 19556342 PMC2754897

[36] Ananthanarayanan M, Li Y, Surapureddi S, Balasubramaniyan N, Ahn J, Goldstein JA, et al Histone H3K4 trimethylation by MLL3 as part of ASCOM complex is critical for NR activation of bile acid transporter genes and is downregulated in cholestasis. Am J Physiol Gastrointest Liver Physiol. 2011;300(5):G771–81. Epub 2011/02/19. 10.1152/ajpgi.00499.2010 21330447 PMC3094144

[37] Lee J, Saha PK, Yang QH, Lee S, Park JY, Suh Y, et al Targeted inactivation of MLL3 histone H3-Lys-4 methyltransferase activity in the mouse reveals vital roles for MLL3 in adipogenesis. Proc Natl Acad Sci USA. 2008;105(49):19229–34. Epub 2008/12/03. 10.1073/pnas.0810100105 19047629 PMC2614744

[38] Kim DH, Rhee JC, Yeo S, Shen R, Lee SK, Lee JW, et al Crucial roles of mixed-lineage leukemia 3 and 4 as epigenetic switches of the hepatic circadian clock controlling bile acid homeostasis in mice. Hepatology. 2015;61(3):1012–23. Epub 2014/10/28. 10.1002/hep.27578 25346535 PMC4474368

[39] Valekunja UK, Edgar RS, Oklejewicz M, van der Horst GT, O'Neill JS, Tamanini F, et al Histone methyltransferase MLL3 contributes to genome-scale circadian transcription. Proc Natl Acad Sci USA. 2013;110(4):1554–9. Epub 2013/01/09. 10.1073/pnas.1214168110 23297224 PMC3557088

[40] Lee J, Kim DH, Lee S, Yang QH, Lee DK, Lee SK, et al A tumor suppressive coactivator complex of p53 containing ASC-2 and histone H3-lysine-4 methyltransferase MLL3 or its paralogue MLL4. Proc Natl Acad Sci USA. 2009;106(21):8513–8. Epub 2009/05/13. 10.1073/pnas.0902873106 19433796 PMC2689008

[41] Lee S, Lee DK, Dou Y, Lee J, Lee B, Kwak E, et al Coactivator as a target gene specificity determinant for histone H3 lysine 4 methyltransferases. Proc Natl Acad Sci USA. 2006;103(42):15392–7. Epub 2006/10/06. 17021013 10.1073/pnas.0607313103 PMC1622834

[42] Kim DH, Kim J, Lee JW. Requirement for MLL3 in p53 regulation of hepatic expression of small heterodimer partner and bile acid homeostasis. Mol Endocrinol. 2011;25(12):2076–83. Epub 2011/10/29. 10.1210/me.2011-1198 22034226 PMC3231838

[43] Burckstummer T, Banning C, Hainzl P, Schobesberger R, Kerzendorfer C, Pauler FM, et al A reversible gene trap collection empowers haploid genetics in human cells. Nat Methods. 2013;10(10):965–71. Epub 2013/10/29. 10.1038/nmeth.2609 24161985 PMC6342250

[44] Li WD, Li QR, Xu SN, Wei FJ, Ye ZJ, Cheng JK, et al Exome sequencing identifies an MLL3 gene germ line mutation in a pedigree of colorectal cancer and acute myeloid leukemia. Blood. 2013;121(8):1478–9. Epub 2013/02/23. 10.1182/blood-2012-12-470559 23429989

[45] Seita J, Sahoo D, Rossi DJ, Bhattacharya D, Serwold T, Inlay MA, et al Gene Expression Commons: an open platform for absolute gene expression profiling. PloS One. 2012;7(7):e40321 Epub 2012/07/21. 10.1371/journal.pone.0040321 22815738 PMC3399844

[46] Lowenberg B, Downing JR, Burnett A. Acute myeloid leukemia. N Engl J Med. 1999;341(14):1051–62. Epub 1999/09/30. 10502596 10.1056/NEJM199909303411407

[47] Elias HK, Schinke C, Bhattacharyya S, Will B, Verma A, Steidl U. Stem cell origin of myelodysplastic syndromes. Oncogene. 2014;33(44):5139–50. Epub 2013/12/18. 10.1038/onc.2013.520 24336326

[48] Rankin EB, Narla A, Park JK, Lin S, Sakamoto KM. Biology of the bone marrow microenvironment and myelodysplastic syndromes. Mol Genet Metab. 2015;116(1–2):24–8. Epub 2015/07/27. 10.1016/j.ymgme.2015.07.004 26210353 PMC4618471

[49] Braun T, Fenaux P. Myelodysplastic Syndromes (MDS) and autoimmune disorders (AD): cause or consequence? Best Prac Res Clin Haematol. 2013;26(4):327–36. Epub 2014/02/11. 10.1016/j.beha.2013.09.003 24507810

[50] Lu Q. The critical importance of epigenetics in autoimmunity. J Autoimmun. 2013;41:1–5. Epub 2013/02/05. 10.1016/j.jaut.2013.01.010 23375849

