# Supplementary figures and images for: Loss of Mll3 Catalytic Function Promotes Aberrant Myelopoiesis
Source: PLoS One. 2016 Sep 9;11(9):e0162515. doi: 10.1371/journal.pone.0162515 (PMC5017600; doi:10.1371/journal.pone.0162515)

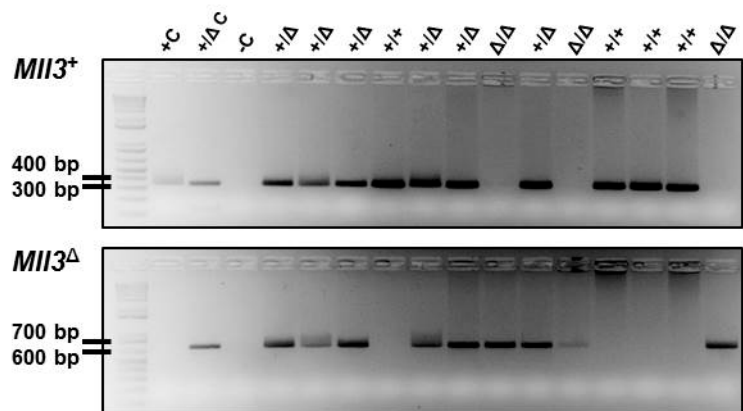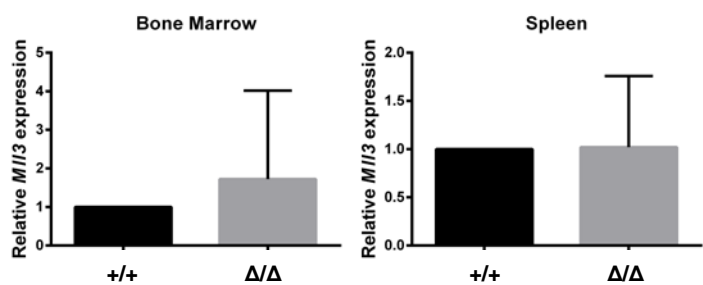

Supplement: S1 Fig — (Top), Tail DNA was analyzed by PCR using primers recognizing either the wild-type allele (+) or the mutant allele (Δ). Products were resolved on an agarose gel, and images were obtained using a Syngene U:Genius3 imaging system (Syngene USA, Frederick, MD). Shown are representative images (top panel, + allele; bottom panel, Δ allele) of specimens used in this study. C, control; -C, no template water control. (Bottom), BM (left) and spleens (right) were harvested from 12-month-old Mll3+/+ and Mll3Δ/Δ mice. Cells were either lineage-depleted (BM) using the EasySep Mouse Hematopoietic Progenitor Cell Isolation Kit, or enriched for Gr1+ cells (spleen) using the EasySep Mouse Neutrophil Enrichment Kit (Stemcell Technologies). qPCR was performed to determine the relative levels of mutant Mll3 expression, normalized to wild-type Mll3, using the following primers: 5’,5’-GTGGCAGAGGCAGGTCTAAA-3’; 3’,5’-GCTGCAAAGTGAACTTGTCACTAC-3’. Left, n = 4 per group; right, n = 1 (Mll3+/+), n = 3 (Mll3Δ/Δ). (PDF) [file pone.0162515.s001.pdf]

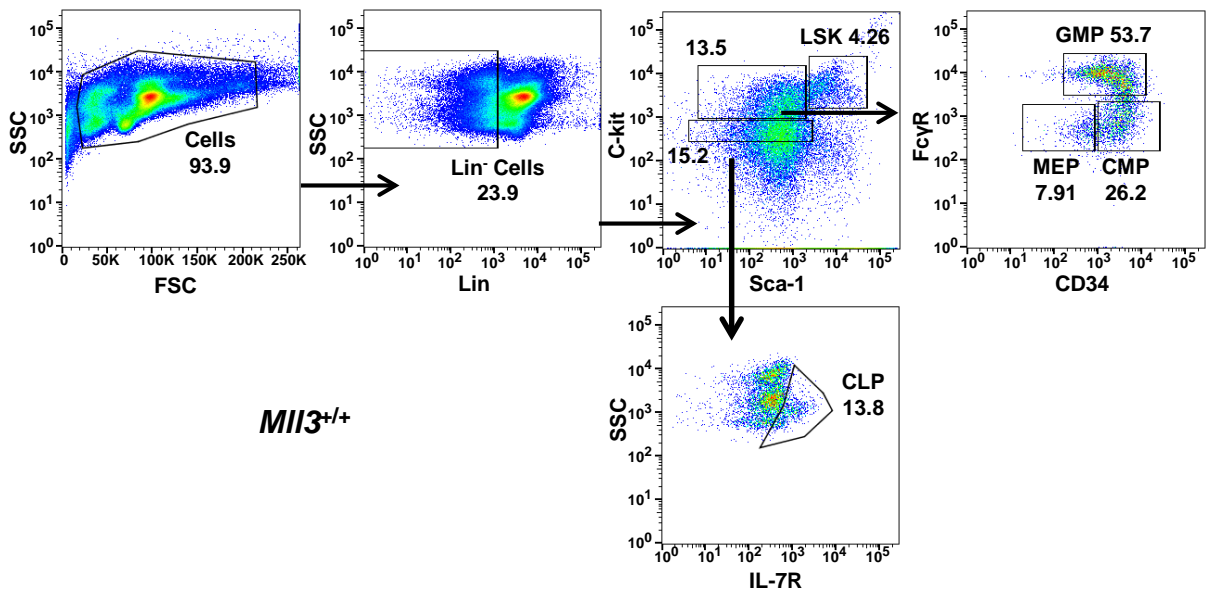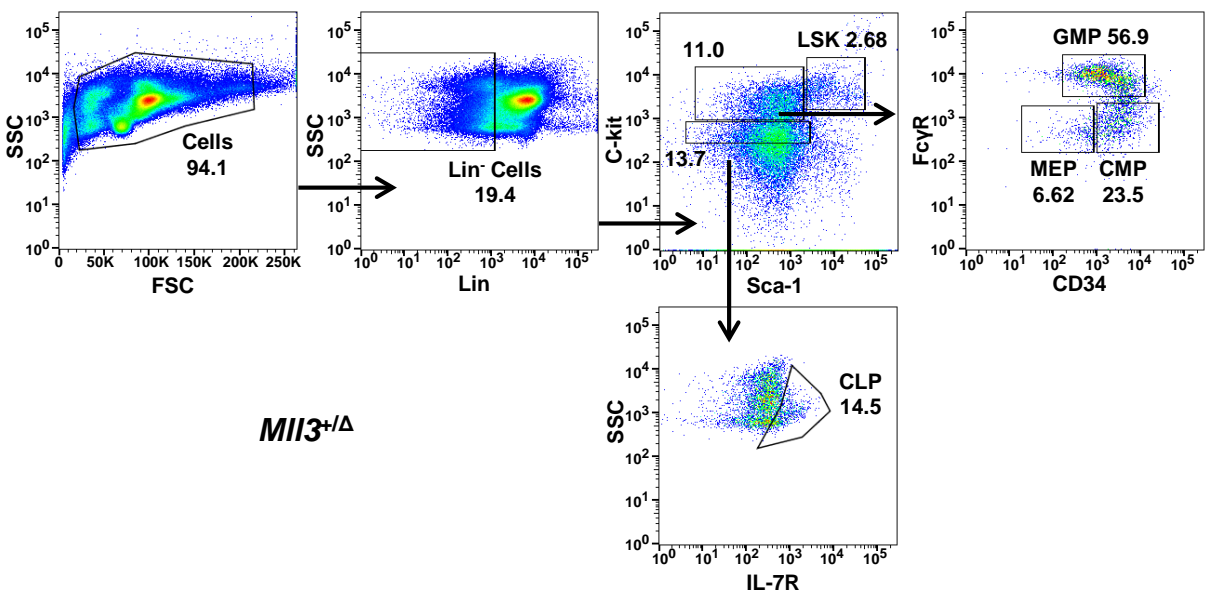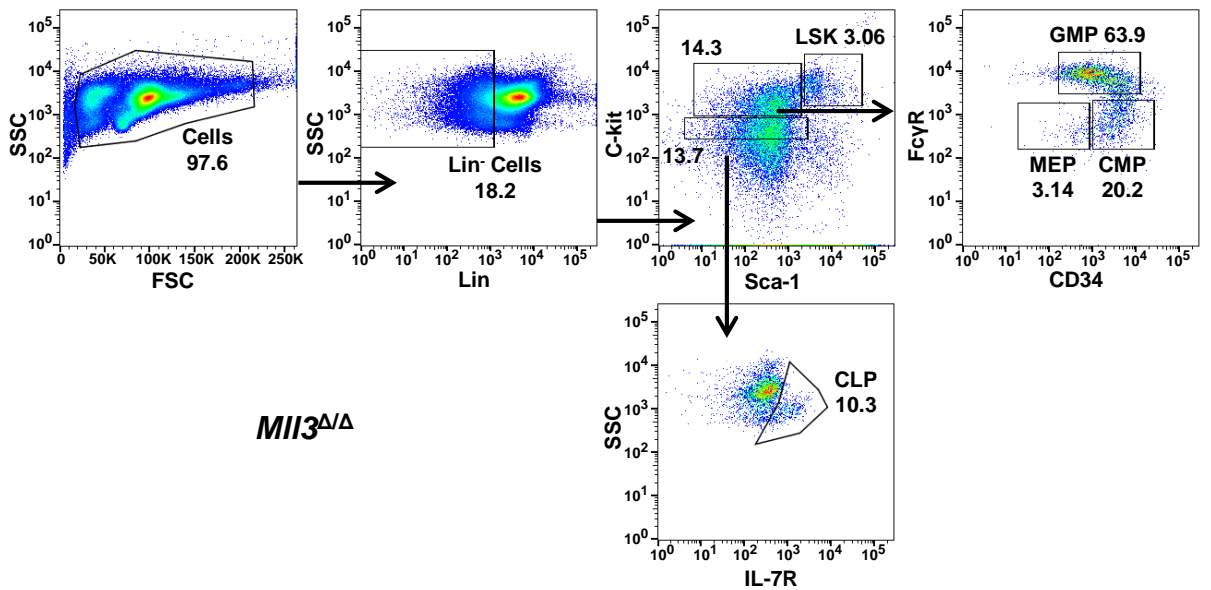

Supplement: S2 Fig — Representative FACS plots are shown for Mll3+/+, Mll3+/Δ, and Mll3Δ/Δ mice. Cells were gated on the Lin- compartment, and then categorized based on c-kit and Sca-1 expression as LSK cells (c-kit+ Sca-1+), CLP cells (c-kitint Sca-1- IL-7R+), or myeloid progenitor cells (c-kit+ Sca-1-), which were further classified as MEP cells (CD34- FcγR-), CMP cells (CD34+ FcγR-), or GMP cells (CD34+ FcγR+). (PDF) [file pone.0162515.s002.pdf]

***MI13<sup>+/+</sup>***

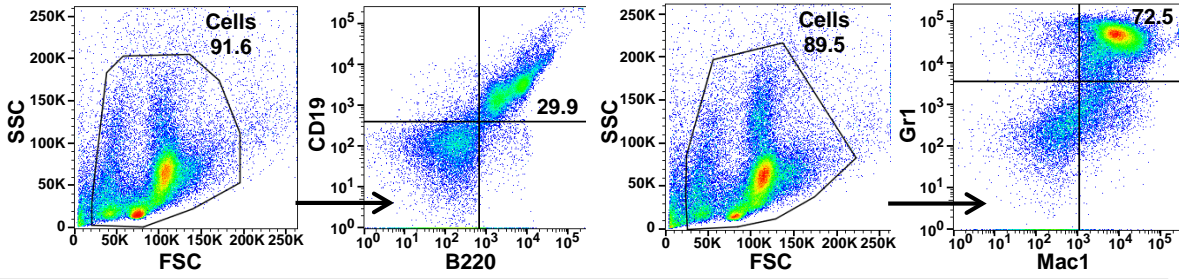

***MI13<sup>+/-</sup>***

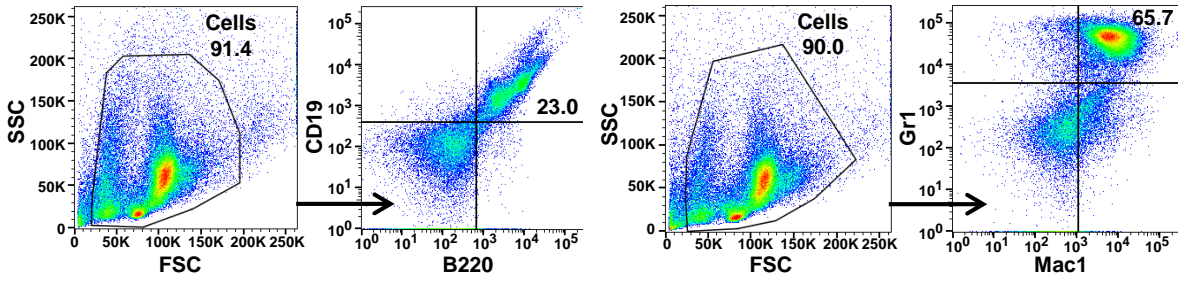

***MI13<sup>Δ/Δ</sup>***

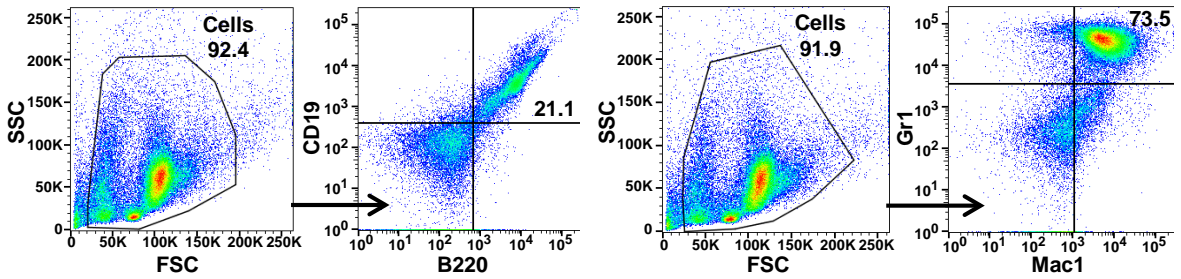

Supplement: S3 Fig — Shown are representative FACS plots for Mll3+/+, Mll3+/Δ, and Mll3Δ/Δ mice. Cells were gated according to CD19 and B220 expression, where B cells were considered positive for both markers, or according to expression of Gr1 and Mac1 (myeloid cells). (PDF) [file pone.0162515.s003.pdf]

***Mll3*<sup>+/ $\Delta$</sup>**   
**(compare to +/+)**

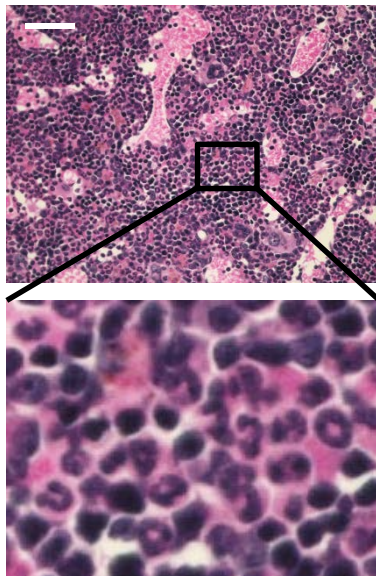

***Mll3*<sup>+/ $\Delta$</sup>**   
**(compare to  $\Delta/\Delta$ )**

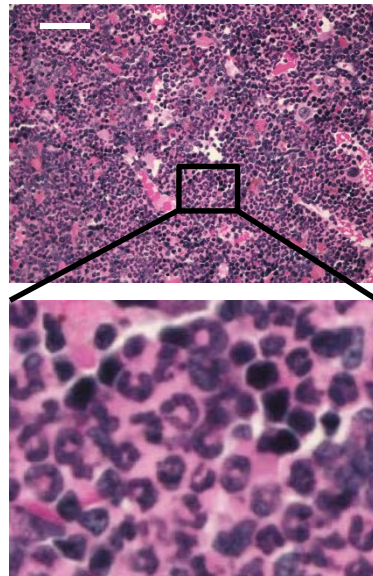

Supplement: S4 Fig — Representative H&E stains from two individual Mll3+/Δ mice of sternum sections taken at 400X magnification. Images show the entire field of view (top panels) and a zoomed-in view (bottom panels). Scale bars are 50μm. Compare to Fig 1D. (PDF) [file pone.0162515.s004.pdf]

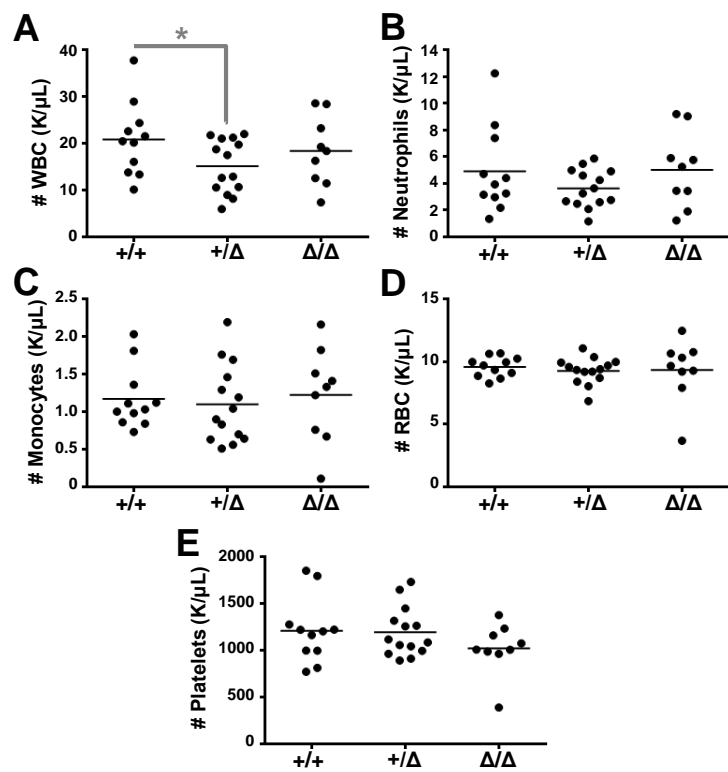

Supplement: S6 Fig — Blood samples were collected via tail nick from 12-month-old mice and run on a Hemavet 950 FS (Drew Scientific, Inc., Miami Lakes, FL) to obtain blood cell counts. Shown is the number of A, white blood cells, B, neutrophils/granulocytes, C, monocytes, D, red blood cells, and E, platelets, where each point represents one mouse. *p<0.05 as determined by the Student’s t-test. (PDF) [file pone.0162515.s006.pdf]

*MI13<sup>+/+</sup>*

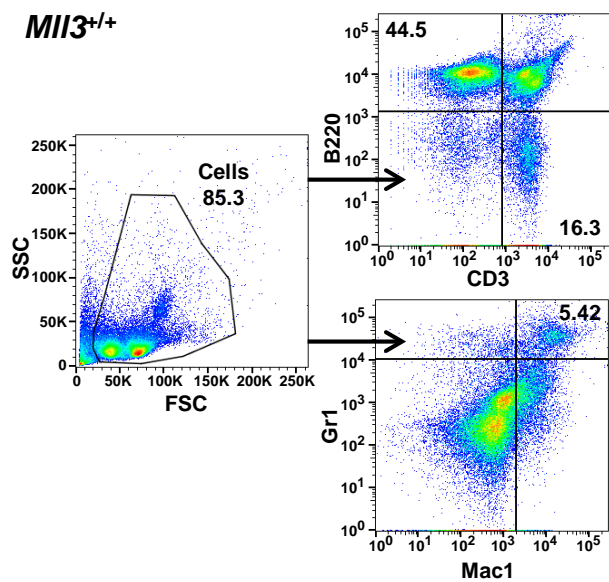

*MI13<sup>+/-</sup>*

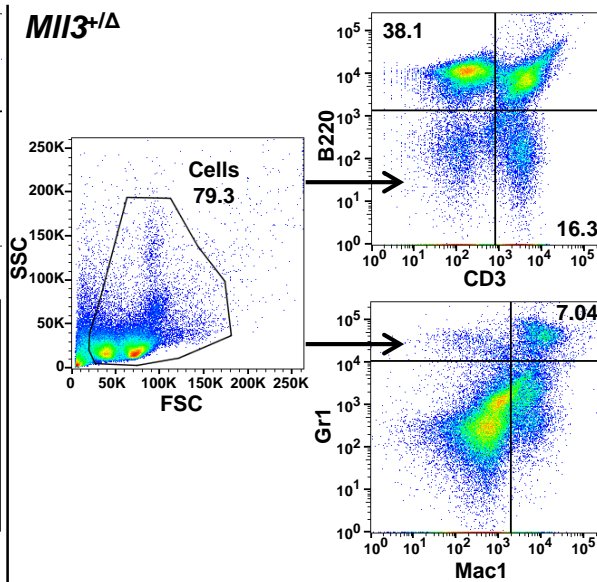

*MI13<sup>Δ/Δ</sup>*

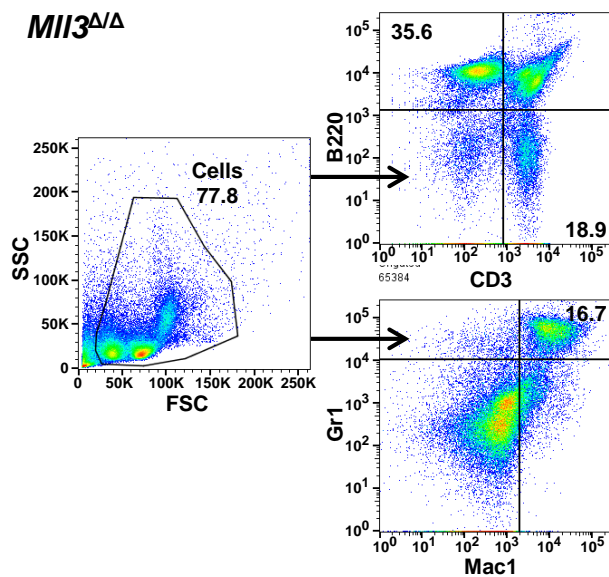

Supplement: S7 Fig — Representative FACS plots are shown for Mll3+/+, Mll3+/Δ, and Mll3Δ/Δ mice. Cells were categorized according to expression of B220 (B cells), CD3 (T cells), or Gr1 and Mac1 (myeloid cells). (PDF) [file pone.0162515.s007.pdf]

***Mll3*<sup>+/ $\Delta$</sup>**   
**(compare to +/+)**

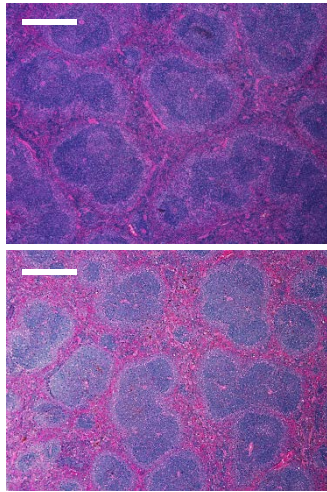

***Mll3*<sup>+/ $\Delta$</sup>**   
**(compare to  $\Delta/\Delta$ )**

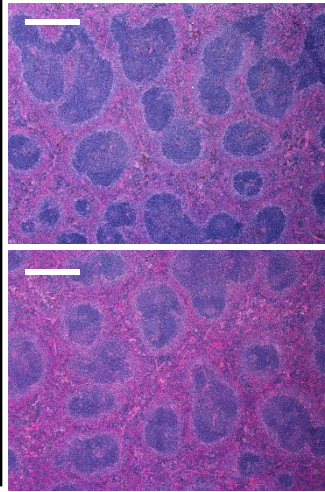

Supplement: S8 Fig — Representative H&E stains of spleen sections taken at 50X magnification from four individual Mll3+/Δ mice. Scale bars are 500μm. Compare to Fig 4D. (PDF) [file pone.0162515.s008.pdf]

***MI13<sup>+/+</sup>***

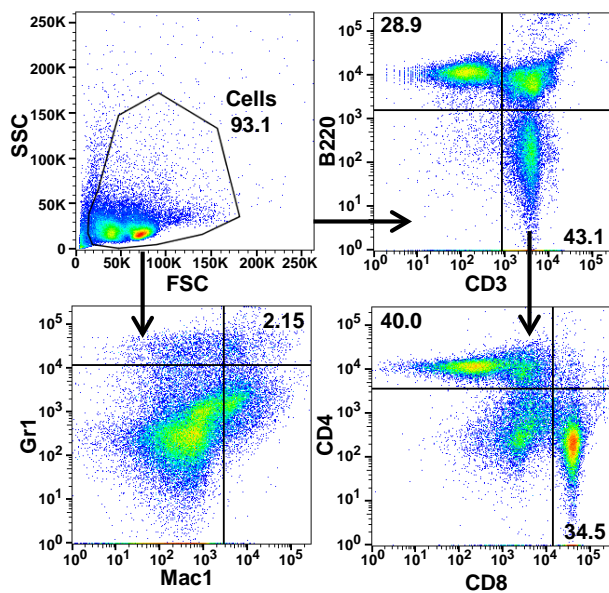

***MI13<sup>+/-</sup>***

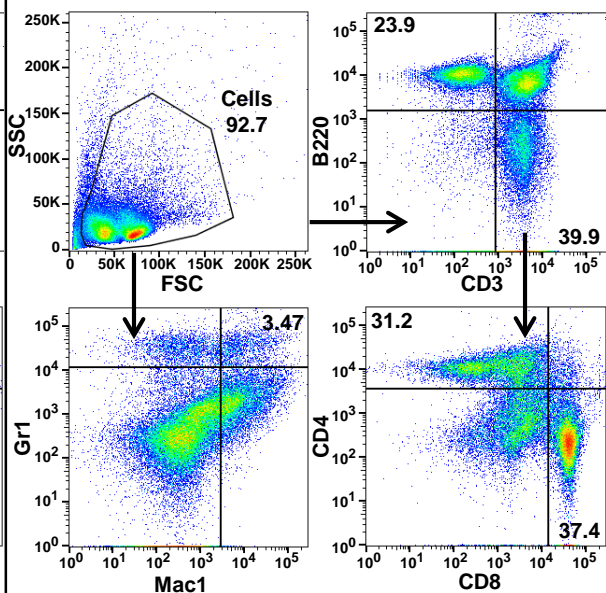

***MI13<sup>Δ/Δ</sup>***

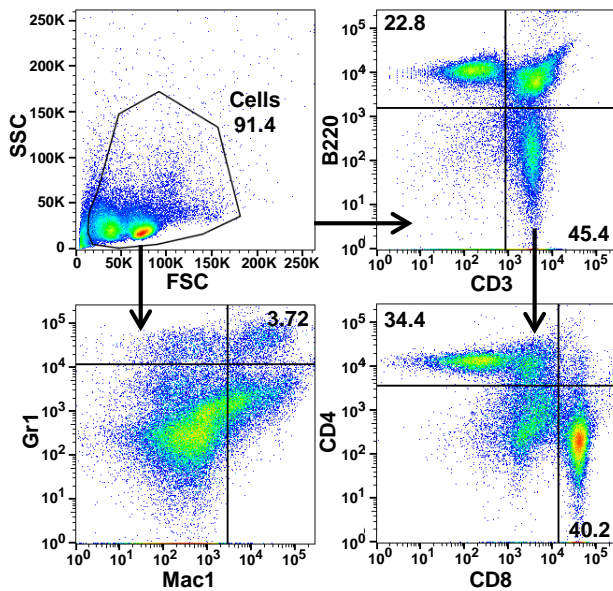

Supplement: S9 Fig — Representative FACS plots for Mll3+/+, Mll3+/Δ, and Mll3Δ/Δ mice. Cells were classified according to expression of B220 (B cells), CD3 (T cells), or Gr1 and Mac1 (myeloid cells). CD3+ cells were further categorized into T cell subsets based on expression of CD4 and CD8. (PDF) [file pone.0162515.s009.pdf]

***Mll3*<sup>+/ $\Delta$</sup>**   
(compare to +/+)

***Mll3*<sup>+/ $\Delta$</sup>**   
(compare to  $\Delta/\Delta$ )

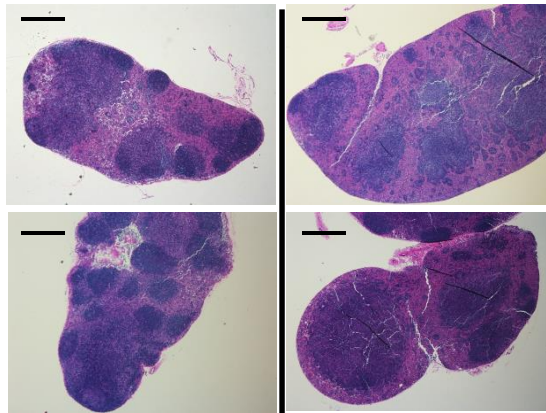

Supplement: S10 Fig — Representative H&E stains from four individual Mll3+/Δ mice of LN sections (50X magnification). Scale bars are 500μm. Compare to Fig 5E. (PDF) [file pone.0162515.s010.pdf]
