# Supplementary material for: Loss of Mll3 Catalytic Function Promotes Aberrant Myelopoiesis
Source: PLoS One. 2016 Sep 9;11(9):e0162515. doi: 10.1371/journal.pone.0162515 (PMC5017600; doi:10.1371/journal.pone.0162515)
Supplement: S5 Fig — Chart depicts Mll3 expression across 39 hematopoietic cell populations in the mouse BM, spleen, and thymus. Pink denotes populations with high Mll3 expression compared to the common reference, whereas blue indicates populations with relatively low Mll3 expression. HSC, hematopoietic stem cell; MPP, multi-potent progenitor; GMLP, granulocyte/macrophage/lymphoid progenitor; MkP, megakaryocyte progenitor; Gra, granulocyte; Mono, monocyte; BLP, B-lymphoid progenitor; Fr, fraction; Mz, marginal zone; Fo, follicular; NK, natural killer cell; DN, double negative; DP, double positive; Plt, platelet; Ery, erythrocyte. (PDF) [file pone.0162515.s005.pdf]

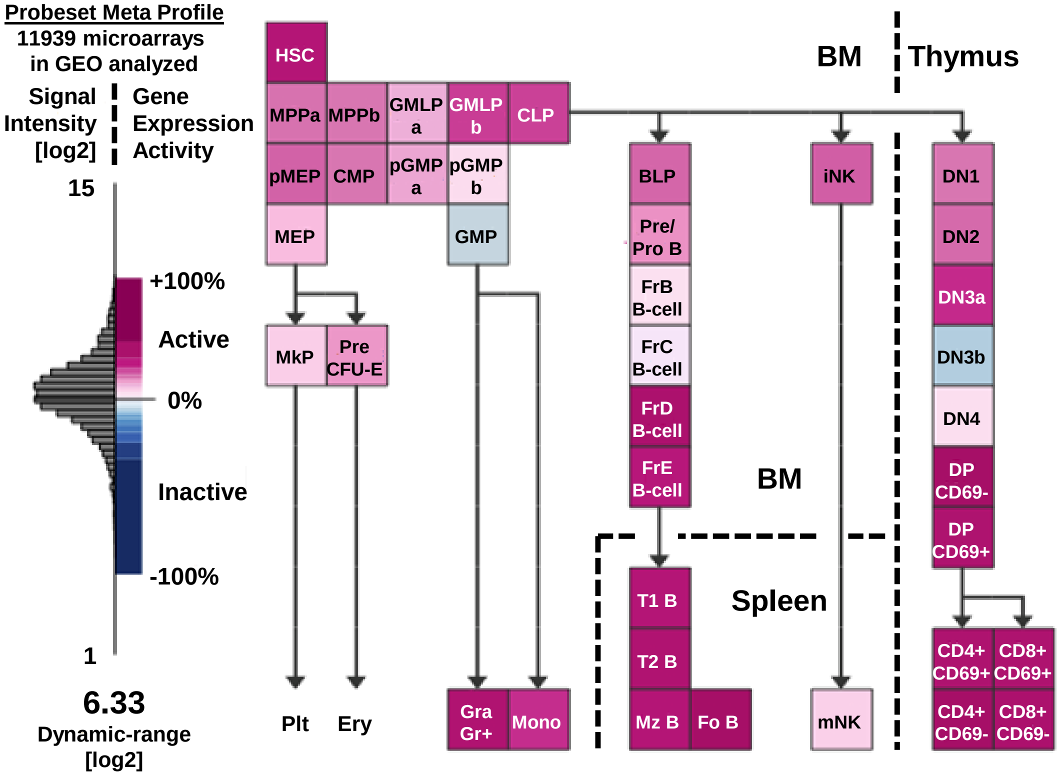
Probeset Meta Profile
11939 microarrays
in GEO analyzed
Signal Intensity [log2]
Gene Expression Activity
15
+100%
Active
0%
Inactive
-100%
1
6.33
Dynamic-range [log2]
BM
Thymus
HSC
MPPa
MPPb
GMLP a
GMLP b
CLP
pMEP
CMP
pGMP a
pGMP b
MEP
GMP
MkP
Pre CFU-E
Plt
Ery
Gra Gr+
Mono
BLP
Pre/ Pro B
FrB B-cell
FrC B-cell
FrD B-cell
FrE B-cell
BM
Spleen
T1 B
T2 B
Mz B
Fo B
iNK
mNK
DN1
DN2
DN3a
DN3b
DN4
DP CD69-
DP CD69+
CD4+ CD69+
CD8+ CD69+
CD4+ CD69-
CD8+ CD69-
